# RSS-Based Method for Sensor Localization with Unknown Transmit Power and Uncertainty in Path Loss Exponent

**DOI:** 10.3390/s16091452

**Published:** 2016-09-08

**Authors:** Jiyan Huang, Peng Liu, Wei Lin, Guan Gui

**Affiliations:** 1 School of Electronic Engineering, University of Electronic Science and Technology of China, Chengdu 610054, China; 201422020706@std.uestc.edu.cn; 2 Institute of Electronic and Information Engineering in Dongguan UESTC, Dongguan 523808, China; 3 Space Star Technology Co., Ltd. and State Key Laboratory of Space-Ground Integrated Information Technology, Beijing 100191, China; lp1381074@space-star.com; 4 Department of Electronics and Information Systems, Akita Prefectural University, Akita 015-0055, Japan; guiguan@akita-pu.ac.jp

**Keywords:** sensor localization, cramer-rao lower bound (CRLB), received signal strength (RSS), transmit power, path loss exponent (PLE)

## Abstract

The localization of a sensor in wireless sensor networks (WSNs) has now gained considerable attention. Since the transmit power and path loss exponent (PLE) are two critical parameters in the received signal strength (RSS) localization technique, many RSS-based location methods, considering the case that both the transmit power and PLE are unknown, have been proposed in the literature. However, these methods require a search process, and cannot give a closed-form solution to sensor localization. In this paper, a novel RSS localization method with a closed-form solution based on a two-step weighted least squares estimator is proposed for the case with the unknown transmit power and uncertainty in PLE. Furthermore, the complete performance analysis of the proposed method is given in the paper. Both the theoretical variance and Cramer-Rao lower bound (CRLB) are derived. The relationships between the deterministic CRLB and the proposed stochastic CRLB are presented. The paper also proves that the proposed method can reach the stochastic CRLB.

## 1. Introduction

Wireless sensor networks (WSNs) have been widely used for monitoring and control in military, environmental, health and commercial systems [1,2,3,4]. A WSN usually consists of tens or hundreds of wirelessly connected sensors. Sensor positioning becomes an important issue. Since the global positioning system (GPS) is currently a costly solution, only a small percentage of sensors are equipped with GPS receivers called reference devices (RDs), whereas the other sensors are blindfolded devices (BDs).

Several geolocation techniques have been used to estimate sensor positions, including the time-of-arrival (TOA)-, the time-difference-of-arrival (TDOA)-, the angle-of-arrival (AOA)-, the received signal strength (RSS)-based methods or hybrid location methods [5,6,7,8]. Among these location techniques, a method based on RSS has attracted much attention because of its low complexity and low cost of devices [9]. For the RSS localization technique, the transmit power and path loss exponent (PLE) are two critical parameters which have significant effects on the positioning accuracy. Many RSS methods and performance analyses have been reported in the literature [10,11,12,13,14,15]. These studies assumed that both the transmit power and PLE are perfectly known. However, this assumption is not suitable for a practical channel environment. Since the transmit power of a sensor in the WSNs depends on its battery, antenna gain, and scheduling algorithm, it is changing with different sensors and times. The additional hardware and software caused by the transmission of the transmit power between RDs and a BD make the WSNs more convoluted. Moreover, the transmit power is usually unknown for non-cooperative applications such as sensor localization in military areas. Compared with the transmit power, the PLE is not only a time-varying parameter but also a function of the channel environment. It changes as the environment and time change. Subsequently, various RSS-based location methods [16,17,18,19,20,21,22] have been proposed to consider the case where both the transmit power and PLE are unknown. Different estimators such as maximum likelihood (ML) and semidefinite programming (SDP) are devised in those algorithms. Although those algorithms can provide the optimum or suboptimum performance, they require a search or alternating process, and cannot give a closed-form solution to sensor localization. The inefficiency incurred by these algorithms may not be feasible to be applied in a practical system. In addition, the performance of the search methods may strongly rely on the initial solution. An improper selection of the initialization may lead to a local minimum and cause a large estimation error. Besides the above localization algorithms, some studies have been reported on performance analyses for sensor localization using RSS measurements [16,23]. The authors in [16] derived the Cramer-Rao lower bound (CRLB) for the RSS location technique considering the case with the unknown transmitted power and PLE. Furthermore, a more practical CRLB was proposed in [23]. Besides the unknown transmitted power and PLE, the derived CRLB in [23] considers both the antenna radiation pattern information and the differences of PLE among RDs. There are deterministic and stochastic CRLBs for the RSS localization technique. The former regards the PLE as a deterministic unknown parameter while the latter models the PLE as a Gaussian random variable. Although the deterministic CRLBs for the RSS localization technique have been addressed in the literature [16,23], the stochastic CRLB of RSS methods has not been studied. This paper will try to derive the stochastic CRLB for RSS-based location methods.

In this paper, a novel RSS localization method based on a two-step weighted least squares (WLS) estimator is proposed for the case with the unknown transmit power and uncertainty in PLE. Furthermore, the complete performance analysis of the proposed method is given in the paper. Since the mobile location is a nonlinear problem, some approximations are essential for the proposed method to obtain the closed-form solution. The perturbation approach retaining only the linear perturbation terms is used here to solve the nonlinear mobile location problem. Theoretical analysis proves that the theoretical variance of the proposed method is equal to the CRLB. This means that the effect of the approximations is extremely small. Compared with the previous research studies, the main contributions of this paper are listed as follows:

(1) The proposed method can not only provide the closed-form solution but it can also attain the CRLB which is verified by the theoretical analysis and simulations. The existing RSS methods considering the unknown transmit power and uncertainty in PLE require a search process.
(2) Compared with the deterministic assumption of PLE in the literature [16,17,18,19,20,21,22,23], the stochastic assumption used in this paper is more suitable for a real environment. The existing RSS methods in [16,17,18,19,20,21,22,23] regarded the PLE as a deterministic unknown parameter and tried to estimate it. These methods assumed that there is no prior knowledge on the PLE. Based on this assumption, the deterministic CRLB is derived in [16,23]. Although it is very hard to obtain an exact value of the PLE, approximate estimates of the PLE for typical channel environments can be obtained through a field test which has been reported in the literature [24,25]. To describe the uncertainty of the PLE, this paper models the PLE as a Gaussian random variable whose mean and variance are treated as prior knowledge and can be obtained from experimental analysis.
(3) The performance analysis for the proposed method is presented in this paper. Both the theoretical variance and stochastic CRLB for the proposed method are derived in the paper. Moreover, some characteristics of the proposed stochastic CRLB for WSN localization are derived in this paper. The relationships between the deterministic CRLB [16] and the proposed stochastic CRLB are presented in Propositions 1 and 2. The paper also proves that the proposed method can reach the stochastic CRLB. These have not been addressed in the literature.

Section 2 briefly introduces the system model. A novel RSS method for the case with the unknown transmit power and uncertainty in the PLE is proposed in Section 3. Section 4 derives the stochastic CRLB. Some characteristics of the proposed methods are given in Section 5. In Section 6, the performance of the proposed algorithm is simulated in terms of the root mean square error (RMSE). The conclusions of this paper are given in Section 7.

## 2. System Model

The basic RSS model is briefly introduced in this section. Assume that (*x*, *y*) is the position of a BD to be estimated and the known coordinate of the *i*th RD in a N-RDs system is (*x_i_*, *y*_i_). Without loss of generality, the position of the first RD can be set to be (0, 0). Denote the measurement with noise of {*} as $\left\{\overset{⌢}{*}\right\}$. The true distance between the *i*th RD and BD can be modeled as:

(1) $$r_{i}^{2}={\left(x_{i}-x\right)}^{2}+{\left(y_{i}-y\right)}^{2}=k_{i}-2x_{i}x-2y_{i}y+k$$

where $k_{i}=x_{i}^{2}+y_{i}^{2}$ and $k=x^{2}+y^{2}$.

Since the measured received power ${\overset{⌢}{P}}_{i}$ at RD i (in decibel milliwatts) can be modeled as a log-normal variable [10], the relation between ${\overset{⌢}{P}}_{i}$ and $r_{i}$ is:

(2) $${\overset{⌢}{P}}_{i}=P_{0}-10\beta \log_{10}\left(\frac{r_{i}}{r_{0}}\right)+n_{i}$$

where *β* is the PLE, $n_{i}$ is a zero-mean Gaussian random process with variance $\sigma^{2}$ in decibels, $P_{0}$ is the reference power at reference distance $r_{0}$ and it depends on the transmit power. Typically, r_0_ = 1 m. Note that Equation (2) is the most popular RSS model and has been widely used in the literature [10,11,12,13,14,15,16,17,18,19,20,21,22,23,24,25]. Moreover, the model has been validated by a variety of measurement results [10,21,25]. For several non-cooperative applications such as mobile location in military areas, the transmit power is usually unknown to RDs which leads to the unknown $P_{0}$. The PLE *β* is a function of the environment and varies typically between 2 (free space) and 4. For particular environments, *β* may be known from experimental analysis. The earlier research [10,11,12,13,14,15] on mobile locations using RSS measurements assumed that *β* can be exactly obtained. This assumption is not suitable for a real situation since *β* changes as the channel environment and time change. To improve the positioning accuracy, several studies [16,17,18,19,20,21,22,23] subsequently regarded the PLE as a deterministic unknown parameter and tried to estimate it. Compared with the deterministic assumption in [16,17,18,19,20,21,22,23], this paper models the PLE as a Gaussian random variable whose mean and variance can be obtained from experimental analysis.

Considering the uncertainty of PLE, *β* in this paper is modeled as:

(3) $$\beta =\beta_{0}+n_{\beta }$$

where *β_0_* is the mean of *β* and can be obtained through the field test, and the disturbance $n_{\beta }$ is used to describe the uncertainty of the PLE, caused by the changes of environment and time, which is assumed to be an independent zero-mean Gaussian distribution with variance $\sigma_{\beta }^{2}$ based on the law of large numbers.

## 3. Closed-Form Solutions

From Equation (2), $r_{i}$ can be obtained:

(4) $$r_{i}=10^{\left(P_{0}-P_{i}\right)/10\beta }$$

Substituting Equation (1) into Equation (4) gives:

$$2x_{i}x+2y_{i}y-k+10^{P_{0}/5\beta }10^{-P_{i}/5\beta }=k_{i}$$

With the RSS noise and PLE disturbance, the error vector derived from Equations (2) and (4) is:

(5) e=Y−GZ

where $G=\left[\begin{array}{cccc} 2x_{1} & 2y_{1} & -1 & 10^{-{\overset{⌢}{P}}_{1}/5\beta }\\ \vdots & \vdots & \vdots & \vdots \\ 2x_{N} & 2y_{N} & -1 & 10^{-{\overset{⌢}{P}}_{N}/5\beta } \end{array}\right]$, $Y=\left[\begin{array}{c} k_{1}\\ \vdots \\ k_{N} \end{array}\right]$, $Z=\left[\begin{array}{c} x\\ y\\ k\\ 10^{P_{0}/5\beta } \end{array}\right]$.

The first step WLS estimator of Z can be obtained from Equation (5):

(6) $$Z=\arg\min\left\{{\left(Y-GZ\right)}^{T}\Psi^{-1}\left(Y-GZ\right)\right\}={\left(G^{T}\Psi^{-1}G\right)}^{-1}G^{T}\Psi^{-1}Y$$

where ψ is the covariance matrix of **e**:

(7) $$\psi =\mathrm{cov}\left(e\right)=E\left(ee^{T}\right)$$

It should be noted that matrix G contains the unknown parameter *β*. To solve Equation (6), the mean *β*_0_ of *β* is used in G. Matrix G becomes:

$$G=\left[\begin{array}{cccc} 2x_{1} & 2y_{1} & -1 & 10^{-{\overset{⌢}{P}}_{1}/5\beta_{0}}\\ \vdots & \vdots & \vdots & \vdots \\ 2x_{N} & 2y_{N} & -1 & 10^{-{\overset{⌢}{P}}_{N}/5\beta_{0}} \end{array}\right]$$

Ignoring the square error term derived from Equation (5), the element $e_{i}$ of **e** can be expressed as:

(8) $$e_{i}=\frac{\ln10}{5\beta_{0}^{2}}10^{\frac{P_{0}-P_{i}}{5\beta_{0}}}\left(\beta_{0}n_{i}+\left(P_{0}-P_{i}\right)n_{\beta }\right)$$

From Equation (8), the expectations of $e_{i}e_{j}$ and $e_{i}^{2}$ can be obtained:

(9) $$\begin{array}{cc} E\left(e_{i}e_{j}\right) & =E\left[\left(\frac{\ln10}{5\beta_{0}^{2}}10^{\frac{P_{0}-P_{i}}{5\beta_{0}}}\right)\left(\frac{\ln10}{5\beta_{0}^{2}}10^{\frac{P_{0}-P_{j}}{5\beta_{0}}}\right)\left(\beta_{0}n_{i}+\left(P_{0}-P_{i}\right)n_{\beta }\right)\left(\beta_{0}n_{j}+\left(P_{0}-P_{j}\right)n_{\beta }\right)\right]\\ & =\left(\frac{\ln10}{5\beta_{0}^{2}}10^{\frac{P_{0}-P_{i}}{5\beta_{0}}}\right)\left(\frac{\ln10}{5\beta_{0}^{2}}10^{\frac{P_{0}-P_{j}}{5\beta_{0}}}\right)\left(P_{0}-P_{i}\right)\left(P_{0}-P_{j}\right)\sigma_{\beta }^{2} \end{array}$$

(10) $$E\left(e_{i}^{2}\right)=E\left[{\left(\frac{\ln10}{5\beta_{0}^{2}}10^{\frac{P_{0}-P_{i}}{5\beta_{0}}}\right)}^{2}{\left(\beta_{0}n_{i}+\left(P_{0}-P_{i}\right)n_{\beta }\right)}^{2}\right]={\left(\frac{\ln10}{5\beta_{0}^{2}}10^{\frac{P_{0}-P_{i}}{5\beta_{0}}}\right)}^{2}\left(\beta_{0}^{2}\sigma^{2}+{\left(P_{0}-P_{i}\right)}^{2}\sigma_{\beta }^{2}\right)$$

Substituting Equations (9) and (10) into Equation (7) gives:

(11) $$\psi =\mathrm{cov}\left(e\right)=E\left(ee^{T}\right)=BQB$$

where

(12) $$B=diag\left\{\left[\begin{array}{ccc} \frac{\ln10}{5\beta_{0}^{2}}10^{\frac{P_{0}-P_{1}}{5\beta_{0}}} & \cdots & \frac{\ln10}{5\beta_{0}^{2}}10^{\frac{P_{0}-P_{N}}{5\beta_{0}}} \end{array}\right]\right\}$$

(13) $$Q=\left[\begin{array}{cccc} \beta_{0}^{2}\sigma^{2}+{\left(P_{0}-P_{1}\right)}^{2}\sigma_{\beta }^{2} & \left(P_{0}-P_{1}\right)\left(P_{0}-P_{2}\right)\sigma_{\beta }^{2} & \cdots & \left(P_{0}-P_{1}\right)\left(P_{0}-P_{N}\right)\sigma_{\beta }^{2}\\ \left(P_{0}-P_{2}\right)\left(P_{0}-P_{1}\right)\sigma_{\beta }^{2} & \beta_{0}^{2}\sigma^{2}+{\left(P_{0}-P_{2}\right)}^{2}\sigma_{\beta }^{2} & \cdots & \left(P_{0}-P_{2}\right)\left(P_{0}-P_{N}\right)\sigma_{\beta }^{2}\\ \vdots & \vdots & \ddots & \vdots \\ \left(P_{0}-P_{N}\right)\left(P_{0}-P_{1}\right)\sigma_{\beta }^{2} & \left(P_{0}-P_{N}\right)\left(P_{0}-P_{2}\right)\sigma_{\beta }^{2} & \cdots & \beta_{0}^{2}\sigma^{2}+{\left(P_{0}-P_{N}\right)}^{2}\sigma_{\beta }^{2} \end{array}\right]$$

Since the covariance matrix ψ depends on the unknown $P_{i}$ and $P_{0}$, further approximation is necessary to make the problem solvable. First, the approximate value ${\overset{⌢}{P}}_{i}$ can be used in ψ to replace $P_{i}$. Second, the approximate estimate of $P_{0}$ can be obtained using the least square (LS) estimator:

(14) $$Z={\left(G^{T}G\right)}^{-1}G^{T}Y$$

(15) $${\overset{⌢}{P}}_{0}=5\beta \log_{10}Z_{4}$$

The covariance matrix of Z can be calculated by using the perturbation approach. Further, Δ is denoted as error perturbation. In the presence of noise and disturbance,

(16) $$G=G_{0}+\Delta G$$

(17) $$Z=Z_{0}+\Delta Z$$

(18) $$Y=Y_{0}$$

Equation (6) can be rewritten as:

(19) $$\left(G^{T}\Psi^{-1}G\right)Z=G^{T}\Psi^{-1}Y$$

Substituting Equations (16)–(18) into Equation (19) gives:

(20) $$\left(\left(G_{0}^{T}+\Delta G^{T}\right)\Psi^{-1}\left(G_{0}+\Delta G\right)\right)\left(Z_{0}+\Delta Z\right)=\left(G_{0}^{T}+\Delta G^{T}\right)\Psi^{-1}Y_{0}$$

Ignoring the square error term, Equation (20) can be simplified as:

(21) $$G_{0}^{T}\Psi^{-1}G_{0}Z_{0}+G_{0}^{T}\Psi^{-1}\Delta GZ_{0}+\Delta G^{T}\Psi^{-1}G_{0}Z_{0}+G_{0}^{T}\Psi^{-1}G_{0}\Delta Z=G_{0}^{T}\Psi^{-1}Y_{0}+\Delta G^{T}\Psi^{-1}Y_{0}$$

Without the noise of ${\overset{⌢}{P}}_{i}$ and the disturbance of the PLE,

(22) $$G_{0}Z_{0}=Y_{0}$$

Substituting Equation (22) into Equation (21) gives:

(23) $$\begin{array}{l} G_{0}^{T}\Psi^{-1}\Delta GZ_{0}=-G_{0}^{T}\Psi^{-1}G_{0}\Delta Z\\ \Delta Z=-{\left(G_{0}^{T}\Psi^{-1}G_{0}\right)}^{-1}G_{0}^{T}\Psi^{-1}\Delta GZ_{0} \end{array}$$

Since $G_{0}Z_{0}=Y_{0}$, Equation (5) implies that:

(24) $$e=Y_{0}-\left(G_{0}+\Delta G\right)Z_{0}=Y_{0}-G_{0}Z_{0}-\Delta GZ_{0}=-\Delta GZ_{0}$$

Substituting Equation (24) into Equation (23) gives:

(25) $$\Delta Z={\left(G_{0}^{T}\Psi^{-1}G_{0}\right)}^{-1}G_{0}^{T}\Psi^{-1}e$$

Substituting Equation (25) into cov(Z), the covariance matrix of Z can be obtained:

(26) $$\begin{array}{cc} \mathrm{cov}\left(Z\right) & =E\left[\Delta Z\Delta Z^{T}\right]={\left(G^{T}\Psi^{-1}G\right)}^{-1}G^{T}\Psi^{-1}E\left[ee^{T}\right]\Psi^{-1}G{\left(G^{T}\Psi^{-1}G\right)}^{-1}\\ & ={\left(G^{T}\Psi^{-1}G\right)}^{-1}G^{T}\Psi^{-1}\Psi \Psi^{-1}G{\left(G^{T}\Psi^{-1}G\right)}^{-1}={\left(G^{T}\Psi^{-1}G\right)}^{-1} \end{array}$$

The estimation accuracy can be further improved using the relationship between *x*, *y* and *k*. The first step of the solution of Z in Equation (6) is based on the assumption of independent *x*, *y* and *k*. However, those parameters are correlated by Equation (1). The results can be revised as follows using the relation of Equation (1):

(27) e′=Y′−G′Z′

where $Y\prime =\left[\begin{array}{c} Z_{1}^{2}\\ Z_{2}^{2}\\ Z_{3} \end{array}\right]$, $G\prime =\left[\begin{array}{cc} 1 & 0\\ 0 & 1\\ 1 & 1 \end{array}\right]$, $Z\prime =\left[\begin{array}{c} x^{2}\\ y^{2} \end{array}\right]$.

Let the estimation errors of *x*, *y* and *k* be $\mu_{1}$, $\mu_{2}$, and $\mu_{3}$. Then the elements of Z become:

(28) $$Z_{1}=x+\mu_{1},\;Z_{2}=y+\mu_{2},\;Z_{3}=k+\mu_{3}$$

Substituting Equation (28) into Equation (27) and ignoring the square error term, the entries of e′ can be expressed as:

(29) $$e\prime_{1}=2x\mu_{1},\;e\prime_{2}=2y\mu_{2},\;e\prime_{3}=\mu_{3}$$

Subsequently, the covariance matrix of e′ is:

(30) $$\Psi \prime =E\left(e\prime e\prime^{T}\right)=B\prime {\left\{\mathrm{cov}\left(Z\right)\right\}}_{\left(1:3\right)\times \left(1:3\right)}B\prime$$

where B′=diag{[2x,2y,1]}. In fact, B′ is unknown as B′ contains the true BD position *x* and *y*. As in Equation (11), B′ can be approximated as $B\prime =diag\left\{\left[2Z_{1},2Z_{2},1\right]\right\}$.

The second step of the WLS solution is:

(31) $$Z\prime ={\left(G\prime^{T}\Psi \prime^{-1}G\prime \right)}^{-1}G\prime^{T}\Psi \prime^{-1}Y\prime$$

Similarly, the covariance matrix of Z′ can be obtained by using the perturbation approach:

(32) $$\mathrm{cov}\left(Z\prime \right)={\left(G\prime^{T}\Psi \prime^{-1}G\prime \right)}^{-1}$$

The position estimation Z′′:

(33) $$Z\prime \prime =sign\left(Z\right)\sqrt{Z\prime }$$

In summary, the steps of the proposed method can be listed as follows:

(1) Estimate ψ through substituting Equations (14) and (15) into Equation (11).
(2) The first weight solution of BD can be obtained through substituting Equation (11) into Equation (6).
(3) The final solution of BD can be obtained from Equation (33).

From the definition of Z′ in Equation (28) and by ignoring the square error term, Z′ can be rewritten as:

(34) $$Z\prime_{1}-x^{2}=2xe_{x},\;Z\prime_{2}-y^{2}=2ye_{y}$$

where $e_{x}$ and $e_{y}$ are the estimation errors of *x*, *y* respectively. The covariance matrix of Z′′ can be obtained from Equation (34):

(35) $$\mathrm{cov}\left(Z\prime \prime \right)=B\prime \prime^{-1}\mathrm{cov}(Z\prime )B\prime \prime^{-1}$$

where $B\prime \prime =diag\left\{\left[\begin{array}{cc} 2x & 2y \end{array}\right]\right\}$.

From Equations (26), (30), (32) and (35), the covariance matrix of Z′′ can be finally obtained:

(36) $$\mathrm{cov}\left(Z\prime \prime \right)={\left(B\prime \prime \mathrm{cov}{\left(Z\prime \right)}^{-1}B\prime \prime \right)}^{-1}={\left(B\prime \prime G\prime^{T}\Psi \prime^{-1}G\prime B\prime \prime \right)}^{-1}={\left(B\prime \prime G\prime^{T}B\prime^{-1}{\left\{\mathrm{cov}{\left(Z\right)}_{\left(1:3\right)\times \left(1:3\right)}\right\}}^{-1}B\prime^{-1}G\prime B\prime \prime \right)}^{-1}$$

## 4. Cramer-Rao Lower Bound

It is well known that the CRLB sets a lower limit for the variance or covariance matrix of any unbiased estimate of unknown parameters [26].

There are deterministic and stochastic CRLBs for RSS location methods. The deterministic CRLB is based on the assumption that the PLE is a deterministic unknown process and was derived in [16,23]. However, the stochastic CRLB for the RSS location technique has not been studied in the literature to the best of our knowledge. The subsection derives the stochastic CRLB for RSS-based algorithms where the PLE is modeled as a temporal Gaussian process whose mean and variance can be obtained from experimental analysis. Studies in [24,25] show that the typical values and variation range of PLEs for different channel environments can be estimated through field tests.

Let $\overset{⌢}{P}={\left[\begin{array}{ccc} {\overset{⌢}{P}}_{1} & \cdots & {\overset{⌢}{P}}_{N} \end{array}\right]}^{T}$ be a RSS measurement vector and a parameter vector θ to be estimated, where θ is ${\left[\begin{array}{cccc} x & y & P_{0} & \beta \end{array}\right]}^{T}$. The CRLB matrix is defined as the inverse of the Fisher information matrix (FIM) $J_{\theta }$:

(37) $$E\left(\left(\overset{⌢}{\theta }-\theta \right){\left(\overset{⌢}{\theta }-\theta \right)}^{T}\right)\geq J_{\theta }^{-1}$$

where $\overset{⌢}{\theta }$ is an estimate of θ.

The FIM is determined by [26]:

(38) $$J_{\theta }=E\left[\frac{\partial \ln f\left(\overset{⌢}{P};\theta \right)}{\partial \theta }{\left(\frac{\partial \ln f\left(\overset{⌢}{P};\theta \right)}{\partial \theta }\right)}^{T}\right]$$

Using the Bayes’ theorem,

(39) $$f\left(\overset{⌢}{P};\theta \right)=f\left(\overset{⌢}{P}|\theta \right)f\left(\theta \right)$$

Substituting Equation (39) into Equation (38) gives

(40) $$\begin{array}{cc} J_{\theta } & =E\left[\frac{\partial \ln f\left(\overset{⌢}{P}|\theta \right)}{\partial \theta }{\left(\frac{\partial \ln f\left(\overset{⌢}{P}|\theta \right)}{\partial \theta }\right)}^{T}\right]+E\left[\frac{\partial \ln f\left(\overset{⌢}{P}|\theta \right)}{\partial \theta }{\left(\frac{\partial \ln f\left(\theta \right)}{\partial \theta }\right)}^{T}\right]\\ & +E\left[\frac{\partial \ln f\left(\theta \right)}{\partial \theta }{\left(\frac{\partial \ln f\left(\overset{⌢}{P}|\theta \right)}{\partial \theta }\right)}^{T}\right]+E\left[\frac{\partial \ln f\left(\theta \right)}{\partial \theta }{\left(\frac{\partial \ln f\left(\theta \right)}{\partial \theta }\right)}^{T}\right] \end{array}$$

From Equation (2), the probability density function (PDF) $f\left(\overset{⌢}{P}|\theta \right)$ can be written as:

(41) $$f\left(\overset{⌢}{P}|\theta \right)=\prod_{i=1}^{N}f\left({\overset{⌢}{P}}_{i}|\theta \right)$$

where

$$f\left({\overset{⌢}{P}}_{i}|\theta \right)=\frac{1}{\sqrt{2\pi }\sigma }\exp\left(-\frac{{\left({\overset{⌢}{P}}_{i}-P_{0}+10\beta \log_{10}\left(r_{i}\right)\right)}^{2}}{2\sigma^{2}}\right)$$

The log of $f\left(\overset{⌢}{P}|\theta \right)$ is:

(42) $$\ln f\left(\overset{⌢}{P}|\theta \right)=\sum_{i=1}^{N}\ln f\left({\overset{⌢}{P}}_{i}|\theta \right)$$

Substituting Equation (42) into $\partial \ln f\left(\overset{⌢}{P}|\theta \right)/\partial \theta_{i}$ gives:

$$\frac{\partial \ln f\left(\overset{⌢}{P}|\theta \right)}{\partial x}=\sum_{i=1}^{N}\left(-\frac{{\overset{⌢}{P}}_{i}-P_{0}+10\beta \log_{10}r_{i}}{\sigma^{2}}\right)\frac{10\beta }{r_{i}\ln10}\frac{x_{i}-x}{r_{i}}$$

$$\frac{\partial \ln f\left(\overset{⌢}{P}|\theta \right)}{\partial y}=\sum_{i=1}^{N}\left(-\frac{{\overset{⌢}{P}}_{i}-P_{0}+10\beta \log_{10}r_{i}}{\sigma^{2}}\right)\frac{10\beta }{r_{i}\ln10}\frac{y_{i}-y}{r_{i}}$$

$$\frac{\partial \ln f\left(\overset{⌢}{P}|\theta \right)}{\partial P_{0}}=\sum_{i=1}^{N}\left(-\frac{{\overset{⌢}{P}}_{i}-P_{0}+10\beta \log_{10}r_{i}}{\sigma^{2}}\right)\left(-1\right)$$

$$\frac{\partial \ln f\left(\overset{⌢}{P}|\theta \right)}{\partial \beta }=\sum_{i=1}^{N}\left(-\frac{{\overset{⌢}{P}}_{i}-P_{0}+10\beta \log_{10}r_{i}}{\sigma^{2}}\right)\left(10\log_{10}\left(r_{i}\right)\right)$$

Since $E\left[{\overset{⌢}{P}}_{i}-P_{0}+10\beta \log_{10}\left(r_{i}\right)\right]=0$, the expectation of $\partial \ln f\left(\overset{⌢}{P}|\theta \right)/\partial \theta_{i}$ is:

(43) $$E\left[\partial \ln f\left(\overset{⌢}{P}|\theta \right)/\partial \theta_{i}\right]=0$$

In the presence of the uncertainty of the PLE, the PDF of the PLE can be obtained from Equation (3):

(44) $$f\left(\theta \right)=\frac{1}{\sqrt{2\pi }\sigma_{\beta }}\exp\left(-\frac{{\left(\overset{⌢}{\beta }-\beta \right)}^{2}}{2\sigma_{\beta }^{2}}\right)$$

Substituting Equation (44) into $\partial \ln f\left(\theta \right)/\partial \theta_{i}$ gives:

(45) $$\frac{\partial \ln f\left(\theta \right)}{\partial \theta_{i}}=\{\begin{array}{c} \begin{array}{cc} 0 & \theta_{i}\neq \beta \end{array}\\ \begin{array}{cc} -\frac{\beta -\beta_{0}}{\sigma_{\beta }^{2}} & \theta_{i}=\beta \end{array} \end{array}$$

It can be easily derived from Equation (45) that:

(46) $$E\left[\partial \ln f\left(\theta \right)/\partial \theta_{i}\right]=0$$

Substituting Equations (43) and (46) into Equation (40) gives:

(47) $$J_{\theta }=E\left[\frac{\partial \ln f\left(\overset{⌢}{P}|\theta \right)}{\partial \theta }{\left(\frac{\partial \ln f\left(\overset{⌢}{P}|\theta \right)}{\partial \theta }\right)}^{T}\right]+E\left[\frac{\partial \ln f\left(\theta \right)}{\partial \theta }{\left(\frac{\partial \ln f\left(\theta \right)}{\partial \theta }\right)}^{T}\right]$$

Substituting Equations (42) and (45) into Equation (47) gives:

(48) $$J_{\theta }=HQ_{s}^{-1}H^{T}+Q_{\theta }$$

where $H=\left[\begin{array}{cccc} \frac{10\beta }{r_{1}\ln10}\frac{x_{1}-x}{r_{1}} & \frac{10\beta }{r_{2}\ln10}\frac{x_{2}-x}{r_{2}} & \cdots & \frac{10\beta }{r_{N}\ln10}\frac{x_{N}-x}{r_{N}}\\ \frac{10\beta }{r_{1}\ln10}\frac{y_{1}-y}{r_{1}} & \frac{10\beta }{r_{2}\ln10}\frac{y_{2}-y}{r_{2}} & \cdots & \frac{10\beta }{r_{N}\ln10}\frac{y_{N}-y}{r_{N}}\\ -1 & -1 & \cdots & -1\\ 10\log_{10}\left(r_{1}\right) & 10\log_{10}\left(r_{2}\right) & \cdots & 10\log_{10}\left(r_{N}\right) \end{array}\right]$, $Q_{s}=diag\left\{\left[\begin{array}{ccc} \sigma^{2} & \cdots & \sigma^{2} \end{array}\right]\right\}$, $Q_{\theta }=diag\left\{\left[\begin{array}{cccc} 0 & 0 & 0 & 1/\sigma_{\beta }^{2} \end{array}\right]\right\}$.

It should be noted that $HQ_{s}^{-1}H^{T}$ is the FIM of the deterministic CRLB in [16].

Finally, the proposed CRLB is derived as:

(49) $$CRLB_{x,y}={\left[{\left(HQ_{s}^{-1}H^{T}+Q_{\theta }\right)}^{-1}\right]}_{2\times 2}$$

The analytical formula of the CRLB can help to reduce the computational complexity and assist the performance analysis. It can be observed from Appendix A that the analytical formula of the derived CRLB is expressed as:

(50) $${\left[{\left(HQ_{s}^{-1}H^{T}+Q_{\theta }\right)}^{-1}\right]}_{2\times 2}={\left(\frac{\ln10}{10\beta }\right)}^{2}{\left[\begin{array}{cc} a & b\\ c & d \end{array}\right]}^{-1}={\left(\frac{\ln10}{10\beta }\right)}^{2}\frac{1}{ad-bc}\left[\begin{array}{cc} d & -b\\ -c & a \end{array}\right]$$

where *a*, *b*, *c* and *d* can be obtained from Equations (A14)–(A17) respectively.

## 5. Performance Analysis

The relationships between the deterministic and stochastic CRLBs are provided in the following propositions.

**Proposition 1.**  *In the RSS localization technique, the deterministic CRLB is higher than the proposed stochastic CRLB.*

(51) $$tr\left\{J_{s}^{-1}\right\}=tr\left\{{\left(HQ_{s}^{-1}H^{T}+Q_{\theta }\right)}^{-1}\right\}\leq tr\left\{J_{d}^{-1}\right\}=tr\left\{{\left(HQ_{s}^{-1}H^{T}\right)}^{-1}\right\}$$

*where $J_{s}$ and $J_{d}$ are the FIMs for stochastic and deterministic CRLBs, respectively.*

**Proof.**  Since $Q_{\theta }$ is a positive semi-definite matrix, Equation (51) holds.□

This proposition shows that the prior knowledge $\sigma_{\beta }^{2}$ of the PLE can help to improve the positioning accuracy.

**Proposition 2.**  The proposed stochastic CRLB reduces to the deterministic CRLB if no prior information on the PLE is given.

**Proof.**  For the case without prior information on the PLE, the variance $\sigma_{\beta }^{2}$ of the PLE will approach infinity. This means $1/\sigma_{\beta }^{2}\to 0$ and $Q_{\theta }\to 0$. Substituting $Q_{\theta }=0$ into Equation (48), $J_{s}=HQ_{s}^{-1}H^{T}+Q_{\theta }=HQ_{s}^{-1}H^{T}=J_{d}$.□

This gives a sanity check for the proposed CRLB.

**Proposition 3.**  *The proposed method can attain its stochastic CRLB:*

(52) $$\mathrm{cov}\left(Z\prime \prime \right)={\left(B\prime \prime G\prime^{T}B\prime^{-1}{\left\{\mathrm{cov}{\left(Z\right)}_{\left(1:3\right)\times \left(1:3\right)}\right\}}^{-1}B\prime^{-1}G\prime B\prime \prime \right)}^{-1}={\left[{\left(HQ_{s}^{-1}H^{T}+Q_{\theta }\right)}^{-1}\right]}_{2\times 2}={\left[J_{\theta }^{-1}\right]}_{2\times 2}$$

**Proof.**  It can be proved from Appendix B. □

Proposition 3 shows that the proposed method can provide an optimum performance.

## 6. Simulation Results

A square region of dimensions 40 m × 40 m is considered for the simulations, where the positions of the BD and RDs are randomly distributed in the square space 0 ≤ *x*, *y* ≤ 40 m.

The RMSEs are defined as $\sqrt{E\left[{\left(x-\overset{⌢}{x}\right)}^{2}+{\left(y-\overset{⌢}{y}\right)}^{2}\right]}$ in the units of m, and are obtained from the average of 5000 independent runs. To compare with the proposed method, the LS localization method Equation (14) is used here due to its closed-form solution. Two search algorithms (ML and SDP) are selected for comparisons. The joint ML estimation of *x*, *y*, $P_{0}$ and *β* can be formulated as [21]:

(53) $$\underset{x,y,P_{0},\beta }{\min}\sum_{i=1}^{N}{\left({\overset{⌢}{P}}_{i}-P_{0}+10\beta \log_{10}\left(r_{i}\right)\right)}^{2}$$

The ML estimator is solved by the MATLAB routine lsqnonlin using the Levenberg-Marquardt method. Obviously, Equation (53) is non-convex which may lead to multiple local minima. This implies that the performance of the ML estimator strongly relies on the initial solution. Besides the ML-LM method, an SDP algorithm given in [15] is also included in the simulations since its object function is convex which means its global minima can be obtained. The MATLAB package CVX [27] is used to solve the SDP algorithm. The solver of CVX is SeDuMi [28]. It should be noted that SDP [15] is generally based on the fact that both the transmit power and PLE are available. A summary of the considered algorithms is given in Table 1.

Comparisons among the deterministic CRLB [16], the proposed stochastic CRLB and the theoretical variance of the proposed method are also given in the simulations.

**Figure 1:**
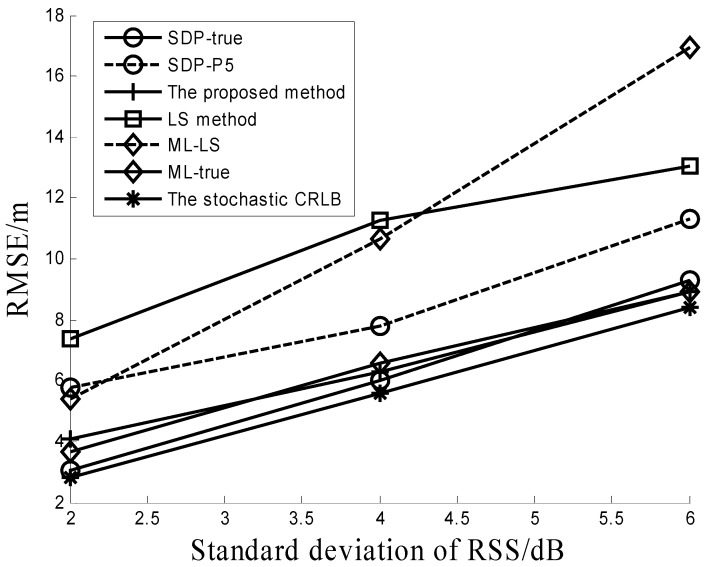
Performance comparison under different RSS noises.

Figure 1 shows the RMSEs versus standard deviations (stds) σ of the RSS measurements when the PLE is equal to 4; then number of RDs is six and the std $\sigma_{\beta }$ of PLE uncertainty is 0.2. It can be seen from the figure that all three methods (the proposed method, ML-TRUE and SDP-TRUE) provide much better performances than the others (LS method, ML-LS and SDP-P5). The performance of the proposed method is similar to those of the ML-TRUE and SDP-TRUE algorithms. It can be observed from Figure 1 that ML-LS performs worse than ML-TRUE. This verifies that the ML estimator depends heavily on its initial solution. Performance comparisons between SDP-P5 and SDP-TRUE in Figure 1 show that a little uncertainty on the transmit power and PLE (5% in simulations) will greatly degrade the performance of the SDP algorithm. It should be noted that both the ML-TRUE and SDP-TRUE algorithms require the true values of the transmit power and PLE, which is not suitable for a real channel situation. Figure 1 also shows that the proposed method can reach its CRLB.

The average running time is compared in Table 2. In the simulation, the PLE is equal to 4, $\sigma_{\beta }=0.2$, σ = 4 dB, the number of RDs is six, and the CPU is i7-2600 3.4 GHZ. It can be seen from Table 2 that the average running times of the ML-LS and SDP-P5 are approximately 60 and 412 times longer than the proposed method. The ML-TRUE has half the running time of the ML-LS since the true initial point may help the ML estimator reach the local minima faster, whereas SDP-TRUE and SDP-P5 have similar running times due to their global minima. As expected, the LS and the proposed method with the closed-form solutions have the shortest running times.

**Figure 2:**
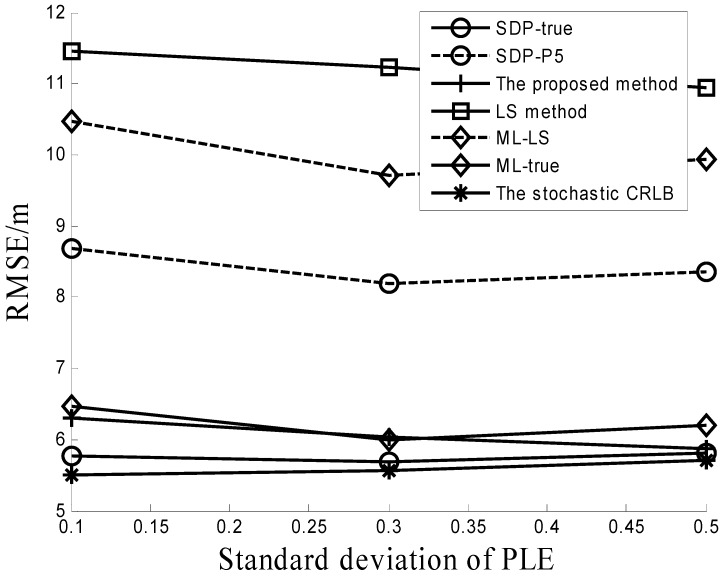
Performance comparison under different PLE uncertainties.

Performance comparisons with different $\sigma_{\beta }$ are recorded in Figure 2. In this simulation, the std $\sigma_{\beta }$ of PLE uncertainty is varied from 0.1 to 0.5, the number of RDs is six, $\beta_{0}=4$, and σ = 4 dB. It can be also observed that the proposed method outperforms the other three algorithms (the LS method, ML-LS and SDP-P5).

**Figure 3:**
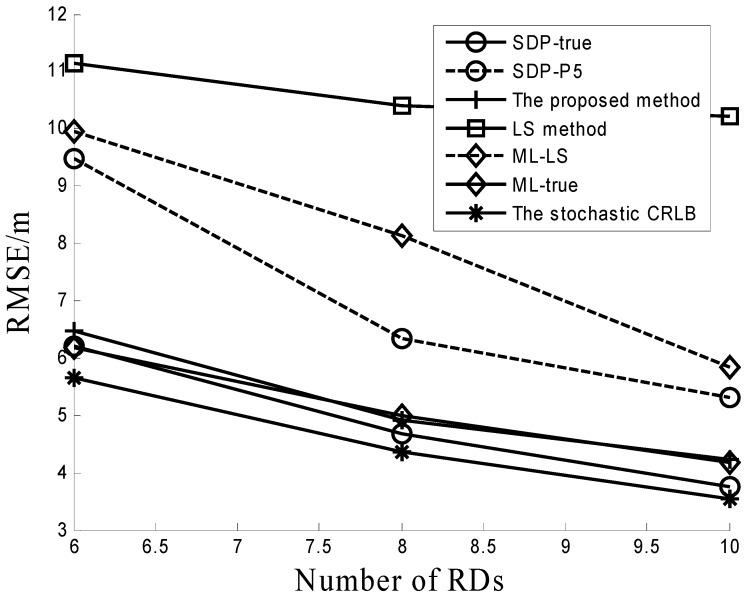
Performance comparison under different numbers of RDs.

The effect of different numbers of RDs is simulated in Figure 3. In this simulation, $\sigma_{\beta }=0.2$, $\beta_{0}=4$, and σ = 4 dB. The number of RDs is varied from six to 10. As the number of RDs increases, the performance of all algorithms becomes better as shown in Figure 3. For various numbers of RDs, the proposed method performs better than other three algorithms (LS method, ML-LS and SDP-P5). This proves the scalability of the proposed method.

**Figure 4:**
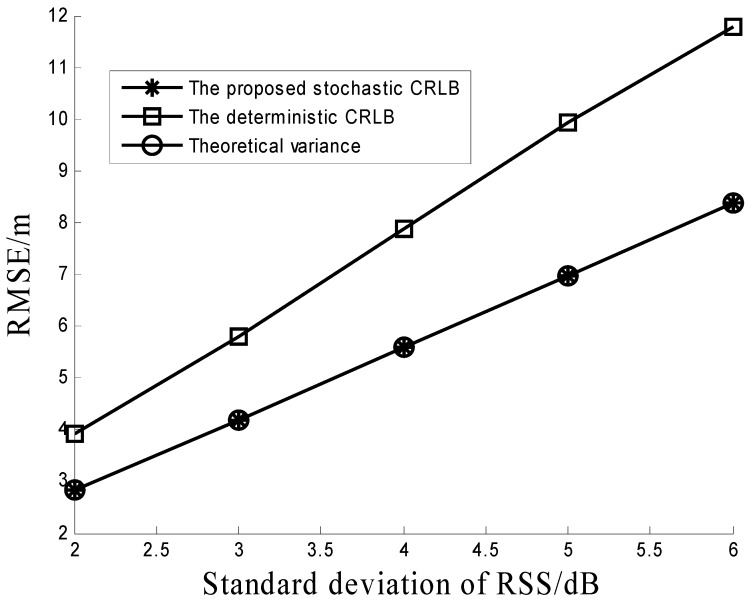
Comparison among the proposed stochastic CRLB, the deterministic CRLB, and the theoretical variance of the proposed method under different RSS noises.

Figure 4 is performed to verify Propositions 1 and 3. The simulation environments of Figure 4 are the same as that of Figure 1. It can be seen from Figure 4 that the theoretical variance of the proposed method is equal to the stochastic CRLB, which means the proposed method can reach its corresponding CRLB. This conclusion matches Proposition 3. Figure 4 also shows that the proposed stochastic CRLB has a better performance than the deterministic CRLB which is in line with Proposition 1. This implies that the positioning accuracy can be improved by the prior information of the PLE.

**Figure 5:**
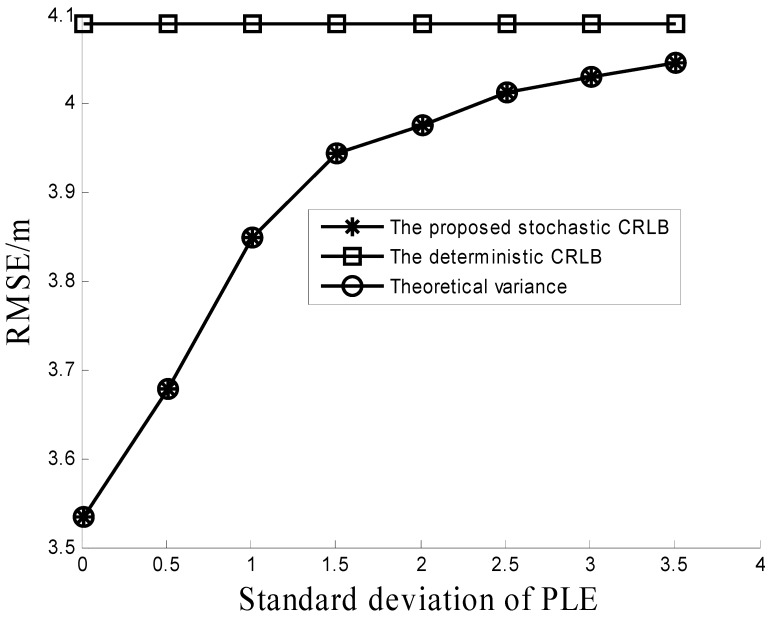
Comparison among the proposed stochastic CRLB, the deterministic CRLB, and the theoretical variance of the proposed method under different PLE uncertainties.

Proposition 2 is evaluated in Figure 5. In this simulation, the std $\sigma_{\beta }$ of the PLE uncertainty is varied from 0.01 to 3.51, the number of RDs is 10, $\beta_{0}=4$, and σ = 4 dB. Figure 5 shows that the stochastic CRLB gradually reaches the deterministic CRLB as the PLE uncertainty $\sigma_{\beta }$ increases. The results mean that the proposed CRLB reduces to the deterministic CRLB if no prior information on the PLE is given, which verifies Proposition 2.

## 7. Conclusions

A novel RSS localization method based on a two-step WLS estimator is proposed for the case with the unknown transmit power and uncertainty in the PLE. Compared with other localization methods, the proposed method can not only provide the closed-form solution but it can also attain the CRLB which is verified by the theoretical analysis and simulations. Both the theoretical variance and stochastic CRLB for the proposed method are derived in the paper. The paper proves that the deterministic CRLB is higher than the proposed stochastic CRLB and the stochastic CRLB reduces to the deterministic CRLB if no prior information on the PLE is given. An experimental proof of the proposed method is left for a future study.

## Figures and Tables

**Table 1:** The summary of the considered algorithms.

Algorithm	Description
LS	The LS estimator in Equation (14) with the closed-form solution
ML-TRUE	The ML estimator in Equation (53) initialized with the true values of the positions of BD, transmit power and PLE
ML-LS	The ML estimator in Equation (53) initialized with the solution of LS
SDP-TRUE	The SDP estimator in [15] initialized with the true transmit powers and PLE
SDP-P5	The SDP estimator in [15] initialized with 5% uncertainty about the transmit power and PLE
The proposed method	The proposed method in Equation (33) with unknown transmit power and PLE

**Table 2:** The average running time of the considered algorithms.

Algorithm	Time (ms)
LS	0.874
ML-TRUE	34.035
ML-LS	59.728
SDP-TRUE	408.806
SDP-P5	407.404
The proposed method	0.988

## Appendix A

To estimate ${\left[J_{\theta }^{-1}\right]}_{2\times 2}$, H and $Q_{\theta }$ are decomposed as:

(A1) $$H={\left[\begin{array}{cc} H_{1}^{T} & H_{2}^{T} \end{array}\right]}^{T},Q_{\theta }=\left[\begin{array}{cc} 0 & 0\\ 0 & Q_{1} \end{array}\right]$$

where $H_{1}=\left[\begin{array}{ccc} \frac{10\beta }{\ln10}\frac{x_{1}-x}{r_{1}^{2}} & \cdots & \frac{10\beta }{\ln10}\frac{x_{N}-x}{r_{N}^{2}}\\ \frac{10\beta }{\ln10}\frac{y_{1}-y}{r_{1}^{2}} & \cdots & \frac{10\beta }{\ln10}\frac{y_{N}-y}{r_{N}^{2}} \end{array}\right],\;H_{2}=\left[\begin{array}{ccc} -1 & \cdots & -1\\ 10\log_{10}\left(r_{1}\right) & \cdots & 10\log_{10}\left(r_{N}\right) \end{array}\right],\;Q_{1}=diag\left\{\left[\begin{array}{cc} 0 & 1/\sigma_{\beta }^{2} \end{array}\right]\right\}$.

Substituting Equation (A1) into Equation (48) gives:

(A2) $$J_{\theta }=\left[\begin{array}{cc} H_{1}Q_{s}^{-1}H_{1}^{T} & H_{1}Q_{s}^{-1}H_{2}^{T}\\ H_{2}Q_{s}^{-1}H_{1}^{T} & H_{2}Q_{s}^{-1}H_{2}^{T}+Q_{1} \end{array}\right]$$

From the matrix inversion lemma [29],

(A3) $${\left[\begin{array}{cc} A_{11} & A_{12}\\ A_{21} & A_{22} \end{array}\right]}^{-1}=\left[\begin{array}{cc} {\left(A_{11}-A_{12}A_{22}^{-1}A_{21}\right)}^{-1} & A_{11}^{-1}A_{12}{\left(A_{21}A_{11}^{-1}A_{12}-A_{22}\right)}^{-1}\\ {\left(A_{21}A_{11}^{-1}A_{12}-A_{22}\right)}^{-1}A_{21}A_{11}^{-1} & {\left(A_{22}-A_{21}A_{11}^{-1}A_{12}\right)}^{-1} \end{array}\right]$$

Using the above equation, ${\left[J_{\theta }^{-1}\right]}_{2\times 2}$ becomes:

(A4) $$\begin{array}{cc} {\left[J_{\theta }^{-1}\right]}_{2\times 2} & ={\left(H_{1}Q_{s}^{-1}H_{1}^{T}-H_{1}Q_{s}^{-1}H_{2}^{T}{\left(H_{2}Q_{s}^{-1}H_{2}^{T}+Q_{1}\right)}^{-1}H_{2}Q_{s}^{-1}H_{1}^{T}\right)}^{-1}\\ & ={\left(H_{1}\left(Q_{s}^{-1}-Q_{s}^{-1}H_{2}^{T}{\left(H_{2}Q_{s}^{-1}H_{2}^{T}+Q_{1}\right)}^{-1}H_{2}Q_{s}^{-1}\right)H_{1}^{T}\right)}^{-1} \end{array}$$

From the second moment matrix inversion formula,

(A5) $${\left[\begin{array}{cc} a & b\\ c & d \end{array}\right]}^{-1}=\frac{1}{ad-bc}\left[\begin{array}{cc} d & -b\\ -c & a \end{array}\right]$$

Then ${\left(H_{2}Q_{s}^{-1}H_{2}^{T}+Q_{1}\right)}^{-1}$ can be expressed as:

(A6) $$\begin{array}{cc} {\left(Q_{1}+H_{2}Q_{s}^{-1}H_{2}^{T}\right)}^{-1} & ={\left[\begin{array}{cc} \frac{N}{\sigma^{2}} & -\frac{1}{\sigma^{2}}\sum_{i=1}^{N}10\log_{10}r_{i}\\ -\frac{1}{\sigma^{2}}\sum_{i=1}^{N}10\log_{10}r_{i} & \frac{1}{\sigma^{2}}\sum_{i=1}^{N}{\left(10\log_{10}r_{i}\right)}^{2}+\frac{1}{\sigma_{\beta }^{2}} \end{array}\right]}^{-1}\\ & =\gamma \left[\begin{array}{cc} \frac{1}{\sigma^{2}}\sum_{i=1}^{N}{\left(10\log_{10}r_{i}\right)}^{2}+\frac{1}{\sigma_{\beta }^{2}} & \frac{1}{\sigma^{2}}\sum_{i=1}^{N}10\log_{10}r_{i}\\ \frac{1}{\sigma^{2}}\sum_{i=1}^{N}10\log_{10}r_{i} & \frac{N}{\sigma^{2}} \end{array}\right] \end{array}$$

where

(A7) $$\gamma =\frac{1}{\frac{N}{\sigma^{2}}\left(\frac{1}{\sigma^{2}}\sum_{i=1}^{N}{\left(10\log_{10}r_{i}\right)}^{2}+\frac{1}{\sigma_{\beta }^{2}}\right)-\frac{1}{\sigma^{4}}{\left(\sum_{i=1}^{N}10\log_{10}r_{i}\right)}^{2}}$$

From Equations (A1) and (A6), we have:

(A8) $$Q_{s}^{-1}H_{2}^{T}{\left(Q_{1}+H_{2}Q_{s}^{-1}H_{2}^{T}\right)}^{-1}H_{2}Q_{s}^{-1}=\gamma C$$

where

(A9) $$\begin{array}{cc} C_{ij} & =-\frac{1}{\sigma^{6}}10\log_{10}r_{i}\sum_{i=1}^{N}10\log_{10}r_{i}+\left(\frac{1}{\sigma^{6}}\sum_{i=1}^{N}{\left(10\log_{10}r_{i}\right)}^{2}+\frac{1}{\sigma^{4}\sigma_{\beta }^{2}}\right)\\ & +\frac{N}{\sigma^{6}}10\log_{10}r_{j}10\log_{10}r_{i}-\frac{1}{\sigma^{6}}10\log_{10}r_{j}\sum_{i=1}^{N}10\log_{10}r_{i} \end{array}$$

Substituting Equation (A8) into Equation (A4) gives:

(A10) $${\left[J_{\theta }^{-1}\right]}_{2\times 2}={\left(H_{1}\left(Q_{s}^{-1}-\gamma C\right)H_{1}^{T}\right)}^{-1}$$

Substituting $H_{1}$ and $Q_{s}$ into $H_{1}Q_{s}^{-1}H_{1}^{T}$ gives:

(A11) $$H_{1}Q_{s}^{-1}H_{1}^{T}={\left(\frac{10\beta }{\sigma \ln10}\right)}^{2}\left[\begin{array}{cc} \sum_{i=1}^{N}\frac{{\left(x_{i}-x\right)}^{2}}{r_{i}^{4}} & \sum_{i=1}^{N}\frac{\left(x_{i}-x\right)\left(y_{i}-y\right)}{r_{i}^{4}}\\ \sum_{i=1}^{N}\frac{\left(x_{i}-x\right)\left(y_{i}-y\right)}{r_{i}^{4}} & \sum_{i=1}^{N}\frac{{\left(y_{i}-y\right)}^{2}}{r_{i}^{4}} \end{array}\right]$$

Substituting Equation (A9) into $\gamma H_{1}CH_{1}^{T}$ gives:

(A12) $$\gamma H_{1}CH_{1}^{T}=\gamma {\left(\frac{10\beta }{\ln10}\right)}^{2}\left[\begin{array}{cc} \sum_{i=1}^{N}\frac{x_{i}-x}{r_{i}^{2}}\sum_{j=1}^{N}C_{ij}\frac{x_{j}-x}{r_{j}^{2}} & \sum_{i=1}^{N}\frac{x_{i}-x}{r_{i}^{2}}\sum_{j=1}^{N}C_{ij}\frac{y_{j}-y}{r_{j}^{2}}\\ \sum_{i=1}^{N}\frac{y_{i}-y}{r_{i}^{2}}\sum_{j=1}^{N}C_{ij}\frac{x_{j}-x}{r_{j}^{2}} & \sum_{i=1}^{N}\frac{y_{i}-y}{r_{i}^{2}}\sum_{j=1}^{N}C_{ij}\frac{y_{j}-y}{r_{j}^{2}} \end{array}\right]$$

From Equations (A11) and (A12), ${\left[J_{\theta }^{-1}\right]}_{2\times 2}$ becomes:

(A13) $${\left[J_{\theta }^{-1}\right]}_{2\times 2}={\left(\frac{\ln10}{10\beta }\right)}^{2}{\left[\begin{array}{cc} a & b\\ c & d \end{array}\right]}^{-1}={\left(\frac{\ln10}{10\beta }\right)}^{2}\frac{1}{ad-bc}\left[\begin{array}{cc} d & -b\\ -c & a \end{array}\right]$$

where

(A14) $$a=\frac{1}{\sigma^{2}}\sum_{i=1}^{N}\frac{{\left(x_{i}-x\right)}^{2}}{r_{i}^{4}}-\gamma \sum_{i=1}^{N}\frac{x_{i}-x}{r_{i}^{2}}\sum_{j=1}^{N}C_{ij}\frac{x_{j}-x}{r_{j}^{2}}$$

(A15) $$b=\frac{1}{\sigma^{2}}\sum_{i=1}^{N}\frac{\left(x_{i}-x\right)\left(y_{i}-y\right)}{r_{i}^{4}}-\gamma \sum_{i=1}^{N}\frac{x_{i}-x}{r_{i}^{2}}\sum_{j=1}^{N}C_{ij}\frac{y_{j}-y}{r_{j}^{2}}$$

(A16) $$c=\frac{1}{\sigma^{2}}\sum_{i=1}^{N}\frac{\left(x_{i}-x\right)\left(y_{i}-y\right)}{r_{i}^{4}}-\gamma \sum_{i=1}^{N}\frac{y_{i}-y}{r_{i}^{2}}\sum_{j=1}^{N}C_{ij}\frac{x_{j}-x}{r_{j}^{2}}$$

(A17) $$d=\frac{1}{\sigma^{2}}\sum_{i=1}^{N}\frac{{\left(y_{i}-y\right)}^{2}}{r_{i}^{4}}-\gamma \sum_{i=1}^{N}\frac{y_{i}-y}{r_{i}^{2}}\sum_{j=1}^{N}C_{ij}\frac{y_{j}-y}{r_{j}^{2}}$$

## Appendix B

It can be seen from Equation (36) that $\mathrm{cov}{\left(Z\right)}_{\left(1:3\right)\times \left(1:3\right)}$ should be obtained firstly to estimate cov(Z′′). To evaluate ${\left\{\mathrm{cov}\left(Z\right)\right\}}_{\left(1:3\right)\times \left(1:3\right)}$, G is decomposed as:

(B1) $$G=\left[\begin{array}{cc} G_{1} & G_{2} \end{array}\right]$$

where

(B2) $$G_{1}=\left[\begin{array}{ccc} 2x_{1} & 2y_{1} & -1\\ \vdots & \vdots & \vdots \\ 2x_{N} & 2y_{N} & -1 \end{array}\right],\;G_{2}=\left[\begin{array}{c} 10^{-P_{1}/5\beta }\\ \vdots \\ 10^{-P_{N}/5\beta } \end{array}\right]=10^{-P_{0}/5\beta }\left[\begin{array}{c} 10^{\left(P_{0}-P_{1}\right)/5\beta }\\ \vdots \\ 10^{\left(P_{0}-P_{N}\right)/5\beta } \end{array}\right]=10^{-P_{0}/5\beta }\left[\begin{array}{c} r_{1}^{2}\\ \vdots \\ r_{N}^{2} \end{array}\right]$$

Substituting Equation (B1) into Equation (26) gives:

(B3) $$\mathrm{cov}\left(Z\right)={\left(G^{T}\Psi^{-1}G\right)}^{-1}={\left[\begin{array}{cc} G_{1}^{T}\psi^{-1}G_{1} & G_{1}^{T}\psi^{-1}G_{2}\\ G_{2}^{T}\psi^{-1}G_{1} & G_{2}^{T}\psi^{-1}G_{2} \end{array}\right]}^{-1}$$

Using the matrix inversion lemma (Equation (A3)), ${\left\{\mathrm{cov}\left(Z\right)\right\}}_{\left(1:3\right)\times \left(1:3\right)}$ becomes:

(B4) $$\begin{array}{cc} {\left\{\mathrm{cov}\left(Z\right)\right\}}_{\left(1:3\right)\times \left(1:3\right)} & ={\left(G_{1}^{T}\psi^{-1}G_{1}-G_{1}^{T}\psi^{-1}G_{2}{\left(G_{2}^{T}\psi^{-1}G_{2}\right)}^{-1}G_{2}^{T}\psi^{-1}G_{1}\right)}^{-1}\\ & ={\left(G_{1}^{T}\left(\psi^{-1}-\psi^{-1}G_{2}{\left(G_{2}^{T}\psi^{-1}G_{2}\right)}^{-1}G_{2}^{T}\psi^{-1}\right)G_{1}\right)}^{-1} \end{array}$$

Substituting Equations (11) and (B4) into Equation (36) gives:

(B5) $$\mathrm{cov}\left(Z\prime \prime \right)=(B\prime \prime G\prime^{T}B\prime^{-1}G_{1}^{T}B^{-1}\left(Q^{-1}-Q^{-1}B^{-1}G_{2}{\left(G_{2}^{T}\psi^{-1}G_{2}\right)}^{-1}G_{2}^{T}B^{-1}Q^{-1}\right){B^{-1}G_{1}B\prime^{-1}G\prime B\prime \prime )}^{-1}$$

Substituting B′′, G′, and B′ into $B\prime \prime G\prime^{T}B\prime^{-1}$ gives:

(B6) $$B\prime \prime G\prime^{T}B\prime^{-1}=\left[\begin{array}{cc} 2x & 0\\ 0 & 2y \end{array}\right]\left[\begin{array}{ccc} 1 & 0 & 1\\ 0 & 1 & 1 \end{array}\right]\left[\begin{array}{ccc} \frac{1}{2x} & 0 & 0\\ 0 & \frac{1}{2y} & 0\\ 0 & 0 & 1 \end{array}\right]=\left[\begin{array}{ccc} 1 & 0 & 2x\\ 0 & 1 & 2y \end{array}\right]$$

Substituting Equation (B6) and $G_{1}$ into $B\prime \prime G\prime^{T}B\prime^{-1}G_{1}^{T}$ gives:

(B7) $$B\prime \prime G\prime^{T}B\prime^{-1}G_{1}^{T}=\left[\begin{array}{ccc} 1 & 0 & 2x\\ 0 & 1 & 2y \end{array}\right]\left[\begin{array}{ccc} 2x_{1} & \cdots & 2x_{N}\\ 2y_{1} & \cdots & 2y_{N}\\ -1 & \cdots & -1 \end{array}\right]=\left[\begin{array}{ccc} 2x_{1}-2x & \cdots & 2x_{N}-2x\\ 2y_{1}-2y & \cdots & 2y_{N}-2y \end{array}\right]$$

It can be seen from Equation (4) that:

(B8) $$r_{i}^{2}=10^{\frac{P_{0}-P_{i}}{5\beta }}$$

(B9) $$P_{0}-P_{i}=10\beta \log_{10}\left(r_{i}\right)$$

Substituting Equations (B8) and (B9) into B and Q gives:

(B10) $$B=diag\left\{\left[\begin{array}{ccc} \frac{\ln10}{5\beta^{2}}r_{1}^{2} & \cdots & \frac{\ln10}{5\beta^{2}}r_{N}^{2} \end{array}\right]\right\}$$

(B11) $$Q=Q_{1}+Q_{2}=\beta^{2}\sigma^{2}I+\left(\sigma_{\beta }\beta A\right){\left(\sigma_{\beta }\beta A\right)}^{T}$$

where

(B12) $$A={\left[\begin{array}{ccc} 10\log_{10}\left(r_{1}\right) & \cdots & 10\log_{10}\left(r_{N}\right) \end{array}\right]}^{T}$$

Using the matrix inversion lemma [29], $Q^{-1}$ can be expressed as:

(B13) $$\begin{array}{cc} Q^{-1} & ={\left(\beta^{2}\sigma^{2}I+\left(\sigma_{\beta }\beta A\right){\left(\sigma_{\beta }\beta A\right)}^{T}\right)}^{-1}\\ & =\frac{1}{\beta^{2}\sigma^{2}}I-\frac{1}{\beta^{2}\sigma^{2}}\left(\sigma_{\beta }\beta A\right){\left(1+\frac{{\left(\sigma_{\beta }\beta A\right)}^{T}\left(\sigma_{\beta }\beta A\right)}{\beta^{2}\sigma^{2}}\right)}^{-1}{\left(\sigma_{\beta }\beta A\right)}^{T}\frac{1}{\beta^{2}\sigma^{2}}\\ & =\frac{1}{\beta^{2}\sigma^{2}}I-\frac{1}{\beta^{2}\sigma^{4}\eta }AA^{T} \end{array}$$

where

(B14) $$\eta =\frac{1}{\sigma_{\beta }^{2}}+\frac{1}{\sigma^{2}}\sum_{i=1}^{N}{\left(10\log_{10}r_{i}\right)}^{2}$$

Substituting Equations (B7) and (B10) into $B\prime \prime G\prime^{T}B\prime^{-1}G_{1}^{T}B^{-1}$ gives:

(B15) $$B\prime \prime G\prime^{T}B\prime^{-1}G_{1}^{T}B^{-1}=\left[\begin{array}{ccc} \frac{\left(x_{1}-x\right)10\beta^{2}}{r_{1}^{2}\ln10} & \cdots & \frac{\left(x_{N}-x\right)10\beta^{2}}{r_{N}^{2}\ln10}\\ \frac{\left(y_{1}-y\right)10\beta^{2}}{r_{1}^{2}\ln10} & \cdots & \frac{\left(y_{N}-y\right)10\beta^{2}}{r_{N}^{2}\ln10} \end{array}\right]$$

It can be observed from Equations (A1) and (B15) that:

(B16) $$B\prime \prime G\prime^{T}B\prime^{-1}G_{1}^{T}B^{-1}=\beta H_{1}$$

Substituting Equations (B16) and (B13) into Equation (B5) gives:

(B17) $$\begin{array}{cc} \mathrm{cov}\left(Z\prime \prime \right) & ={\left(H_{1}\left(\beta^{2}Q^{-1}-\beta^{2}Q^{-1}B^{-1}G_{2}{\left(G_{2}^{T}\psi^{-1}G_{2}\right)}^{-1}G_{2}^{T}B^{-1}Q^{-1}\right)H_{1}^{T}\right)}^{-1}\\ & ={\left(H_{1}\left(Q_{s}^{-1}-\left(\frac{1}{\sigma^{4}\eta }AA^{T}+\frac{P}{\beta^{2}G_{2}^{T}\psi^{-1}G_{2}}\right)\right)H_{1}^{T}\right)}^{-1} \end{array}$$

where

(B18) $$\begin{array}{cc} P & =\left(\frac{1}{\sigma^{2}}I-\frac{1}{\sigma^{4}\eta }AA^{T}\right)B^{-1}G_{2}G_{2}^{T}B^{-1}\left(\frac{1}{\sigma^{2}}I-\frac{1}{\sigma^{4}\eta }AA^{T}\right)\\ & =\frac{1}{\sigma^{4}}B^{-1}G_{2}G_{2}^{T}B^{-1}-\frac{1}{\sigma^{6}\eta }B^{-1}G_{2}G_{2}^{T}B^{-1}AA^{T}-\frac{1}{\sigma^{6}\eta }AA^{T}B^{-1}G_{2}G_{2}^{T}B^{-1}+\frac{1}{\sigma^{8}\eta^{2}}AA^{T}B^{-1}G_{2}G_{2}^{T}B^{-1}AA^{T} \end{array}$$

Substituting $G_{2}$ and Equation (B10) into $G_{2}^{T}B^{-1}$ gives:

(B19) $$G_{2}^{T}B^{-1}=10^{\frac{-P_{0}}{5\beta }}\frac{5\beta^{2}}{\ln10}\left[\begin{array}{ccc} 1 & \cdots & 1 \end{array}\right]$$

Similarly, $AA^{T}$ becomes:

(B20) $$\begin{array}{l} AA^{T}=\\ \left[\begin{array}{ccc} {\left(10\log_{10}r_{1}\right)}^{2} & \cdots & 10\log_{10}r_{1}10\log_{10}r_{N}\\ \vdots & \ddots & \vdots \\ 10\log_{10}r_{N}10\log_{10}r_{1} & \cdots & {\left(10\log_{10}r_{N}\right)}^{2} \end{array}\right] \end{array}$$

Substituting $G_{2}$ and Equation (11) into $G_{2}^{T}\psi^{-1}G_{2}$ gives:

(B21) $$\begin{array}{cc} G_{2}^{T}\psi^{-1}G_{2} & =G_{2}^{T}B^{-1}Q^{-1}B^{-1}G_{2}\\ & =G_{2}^{T}B^{-1}\left(\frac{1}{\beta^{2}\sigma^{2}}I-\frac{1}{\beta^{2}\sigma^{4}\eta }AA^{T}\right)B^{-1}G_{2}\\ & =\frac{1}{\sigma^{2}\beta^{2}}G_{2}^{T}B^{-1}B^{-1}G_{2}-\frac{1}{\beta^{2}\sigma^{4}\eta }G_{2}^{T}B^{-1}AA^{T}B^{-1}G_{2} \end{array}$$

where

(B22) $$G_{2}^{T}B^{-1}B^{-1}G_{2}={\left(10^{\frac{-P_{0}}{5\beta }}\frac{5\beta^{2}}{\ln10}\right)}^{2}N$$

(B23) $$G_{2}^{T}B^{-1}AA^{T}B^{-1}G_{2}={\left(10^{\frac{-P_{0}}{5\beta }}\frac{5\beta^{2}}{\ln10}\right)}^{2}{\left(\sum_{i=1}^{N}10\log_{10}r_{i}\right)}^{2}$$

Substituting Equations (B22) and (B23) into Equation (B21) gives:

(B24) $$G_{2}^{T}\psi^{-1}G_{2}=\frac{N}{\sigma^{2}\beta^{2}}{\left(10^{\frac{-P_{0}}{5\beta }}\frac{5\beta^{2}}{\ln10}\right)}^{2}-\frac{1}{\beta^{2}\sigma^{4}\eta }{\left(10^{\frac{-P_{0}}{5\beta }}\frac{5\beta^{2}}{\ln10}\right)}^{2}{\left(\sum_{i=1}^{N}10\log_{10}r_{i}\right)}^{2}$$

From Equations (B19) and (B20) we have:

(B25) $$B^{-1}G_{2}G_{2}^{T}B^{-1}={\left(10^{\frac{-P_{0}}{5\beta }}\frac{5\beta^{2}}{\ln10}\right)}^{2}1_{N\times N}$$

(B26) $$B^{-1}G_{2}G_{2}^{T}B^{-1}AA^{T}={\left(10^{\frac{-P_{0}}{5\beta }}\frac{5\beta^{2}}{\ln10}\right)}^{2}\sum_{i=1}^{N}10\log_{10}\left(r_{i}\right)\left[\begin{array}{ccc} 10\log_{10}\left(r_{1}\right) & \cdots & 10\log_{10}\left(r_{N}\right)\\ \vdots & \ddots & \vdots \\ 10\log_{10}\left(r_{1}\right) & \cdots & 10\log_{10}\left(r_{N}\right) \end{array}\right]$$

(B27) $$AA^{T}B^{-1}G_{2}G_{2}^{T}B^{-1}={\left(B^{-1}G_{2}G_{2}^{T}B^{-1}AA^{T}\right)}^{T}$$

(B28) $$AA^{T}B^{-1}G_{2}G_{2}^{T}B^{-1}AA^{T}={\left(10^{\frac{-P_{0}}{5\beta }}\frac{5\beta^{2}}{\ln10}\right)}^{2}{\left(\sum_{i=1}^{N}10\log_{10}r_{i}\right)}^{2}\left[\begin{array}{ccc} {\left(10\log_{10}r_{1}\right)}^{2} & \cdots & 10\log_{10}r_{1}10\log_{10}r_{N}\\ \vdots & \ddots & \vdots \\ 10\log_{10}r_{N}10\log_{10}r_{1} & \cdots & {\left(10\log_{10}r_{N}\right)}^{2} \end{array}\right]$$

Substituting Equations (B24)–(B28) into Equation (B18) gives:

(B29) $$\begin{array}{cc} {\left[\frac{P}{\beta^{2}G_{2}^{T}\psi^{-1}G_{2}}\right]}_{ij} & =\gamma (\frac{\eta }{\sigma^{4}}-\frac{1}{\sigma^{6}}\sum_{i=1}^{N}10\log_{10}\left(r_{i}\right)10\log_{10}\left(r_{j}\right)-\frac{1}{\sigma^{6}}\sum_{i=1}^{N}10\log_{10}\left(r_{i}\right)10\log_{10}\left(r_{i}\right)\\ & +\frac{1}{\sigma^{8}\eta }{\left(\sum_{i=1}^{N}10\log_{10}\left(r_{i}\right)\right)}^{2}\left(10\log_{10}r_{i}\right)\left(10\log_{10}r_{j}\right)) \end{array}$$

From Equations (A7), (B14) and (B20), we have:

(B30) $$\begin{array}{cc} {\left[\frac{1}{\sigma^{4}\eta }AA^{T}\right]}_{ij} & =\gamma \left(\frac{1}{\sigma^{4}\eta \gamma }\left(10\log_{10}r_{i}\right)\left(10\log_{10}r_{j}\right)\right)\\ & =\gamma (\frac{N}{\sigma^{6}}\left(10\log_{10}r_{i}\right)\left(10\log_{10}r_{j}\right)-\frac{1}{\sigma^{8}\eta }\left(10\log_{10}r_{i}\right)\left(10\log_{10}r_{j}\right){\left(\sum_{i=1}^{N}10\log_{10}r_{i}\right)}^{2}) \end{array}$$

From Equations (B29) and (B30), we have:

(B31) $$\begin{array}{cc} {\left[\frac{1}{\sigma^{4}\eta }AA^{T}+\frac{P}{\beta^{2}G_{2}^{T}\psi^{-1}G_{2}}\right]}_{ij} & =\gamma (\frac{N}{\sigma^{6}}\left(10\log_{10}r_{i}\right)\left(10\log_{10}r_{j}\right)\\ & \frac{\eta }{\sigma^{4}}-\frac{1}{\sigma^{6}}\sum_{i=1}^{N}10\log_{10}r_{i}10\log_{10}r_{j}-\frac{1}{\sigma^{6}}\sum_{i=1}^{N}10\log_{10}r_{i}10\log_{10}r_{i})\\ & =\gamma C_{ij} \end{array}$$

Substituting (B31) into (B17) gives:

(B32) $${\left\{\mathrm{cov}\left(Z\right)\right\}}_{\left(1:3\right)\times \left(1:3\right)}={\left(H_{1}\left(Q_{s}^{-1}-\gamma C\right)H_{1}^{T}\right)}^{-1}={\left[J_{\theta }^{-1}\right]}_{2\times 2}$$
